# Human Adenovirus Subtype 21a Isolates From Children With Severe Lower Respiratory Illness in China

**DOI:** 10.3389/fmicb.2022.924172

**Published:** 2022-06-16

**Authors:** Wenkuan Liu, Li Zhang, Yong Cai, Qiong Zhang, Dehui Chen, Shuyan Qiu, Yanqun Wang, Duo Xu, Shujun Gu, Xiao Li, Jing Dai, Qian Liu, Rong Zhou, Xingui Tian

**Affiliations:** ^1^State Key Laboratory of Respiratory Diseases, National Clinical Research Center for Respiratory Disease, Guangdong-Hong Kong-Macao Joint Laboratory of Respiratory Infectious Disease, The First Affiliated Hospital of Guangzhou Medical University, Guangzhou Institute of Respiratory Health Guangzhou Medical University, Guangzhou, China; ^2^Guangzhou Laboratory, Guangzhou, China; ^3^Scientific Research Center, The First Affiliated Hospital of Guangdong Pharmaceutical University, Guangzhou, China

**Keywords:** human adenovirus type 21, severe lower respiratory illness, neutralization antigen, variation, severe pneumonia, subtype 21a

## Abstract

Human adenovirus type 21 (HAdV-21) is an important pathogen associated with acute respiratory infection (ARI), but it was rarely reported and characterized so far. In this study, 151 of 1,704 (8.9%) pediatric patients (≤14 years old) hospitalized with ARI in Guangzhou, China in 2019 were positive for HAdV which was the third most frequently detected pathogen. Two HAdV-21-positive patients presented with severe lower respiratory illness and had similar initial symptoms at onset of illness. Then two HAdV-21 strains were isolated and characterized. The two HAdV-21 strains were sequenced and classified as subtype 21a with genomes closely related to strain BB/201903 found in Bengbu, China in March 2019. Phylogenetic analysis for whole genome and major antigen proteins of global HAdV-21 strains showed that HAdV-21 could be classified into two branches, branch 1 including genotype 21p, branch 2 including all other strains dividing into genotype 21a and 21b. There was no significant difference in the plaque size, or the replication curves between the two HAdV-21a strains and the prototype strain HAdV-21p AV-1645. However, there were five highly variable regions (HVR1, HVR3, HVR4, HVR5, and HVR7) in the hexon protein that varied between two branches. Mice immunized with one branch strain showed 2–4-fold lower neutralizing antibody titers against another branch strain. In summary, this study firstly reported two HAdV-21a infections of children in China, characterized two isolates of HAdV-21a associated with severe lower respiratory illness; our results could be important for understanding the HAdV-21 epidemiology and pathogenic, and for developing HAdV-21 vaccine and drug.

## Introduction

Human adenoviruses (HAdVs) are non-enveloped, double-stranded DNA viruses of the family *Adenoviridae*. More than 100 genotypes of HAdVs have been identified, which are classified into seven species (A–G) (Ismail et al., 2018; Ji et al., 2021). HAdVs are associated with a broad spectrum of clinical diseases, such as acute respiratory illness (ARI), conjunctivitis, gastrointestinal infections, and obesity (Sandkovsky et al., 2014; Radke and Cook, 2018; Chen et al., 2020; Ji et al., 2021). Members of species B are known to cause human diseases, with HAdV types 3, 7, 14, and 55 being the most common causes of respiratory disease outbreaks (Prusinkiewicz and Mymryk, 2019).

Human adenovirus type 21 is a member of species B, and was first isolated in 1956 from a 1-year-old child with trachoma and conjunctivitis in Saudi Arabia (Bell et al., 1959). HAdV-21 was later found to be associated with a variety of diseases, including ARI (Becroft, 1971; Darougar et al., 1978; James et al., 1979; Wright et al., 1979; Lee et al., 2018). Severe pneumonia, myocarditis, flaccid paralysis, and even fatal infections in both pediatric and adult patients have been reported (Lang et al., 1969; Henson and Mufson, 1971; Ooi et al., 2003; Hage et al., 2014). The circulation of ARI-associated HAdV-21 has been reported among military recruits and civilians in several developed countries (Pereira, 1963; van der Veen et al., 1969; James et al., 1979; van der Avoort et al., 1986), and caused nosocomial infections in lung transplant patients at a large tertiary care hospital (Philo et al., 2018). However, data on HAdV-21 are limited as only few cases of HAdV-21 infection were reported in North America and Europe (Pfortmueller et al., 2019). HAdV-21 infection reports are especially rare in China (Deng et al., 2007; Ye et al., 2020). To better understand the epidemiology of HAdV-21, infection data from different regions are of great importance.

In this study, we analyzed the distribution of HAdV-21 in pediatric patients with ARI in Guangzhou, China, in 2019. The clinical features of these infected patients, and the characterization of two HAdV-21 strains *in vitro* were described. We also analyzed the antigenic variability among subtypes of human adenovirus 21.

## Materials and Methods

### Respiratory Sample Collection

Respiratory samples, including throat swabs, sputum, and bronchoalveolar lavage fluid, from pediatric patients (≤14 years old) hospitalized with ARI were collected for routine screening of respiratory viruses, *Mycoplasma pneumoniae* (MP) and *Chlamydophila pneumoniae* (CP) in accordance with established clinical protocols at the First Affiliated Hospital of Guangzhou Medical University and The First Affiliated Hospital of Guangdong Pharmaceutical University between January and December 2019 (Liu et al., 2014). The samples were refrigerated at 2–8°C in viral transport medium, transported on ice to the State Key Laboratory of Respiratory Diseases, and analyzed immediately or stored at −80°C before analysis, as previously described (Liu et al., 2011). The patients’ clinical presentations were collected from the medical records.

### Screening for Human Adenovirus and Common Respiratory Pathogens

Respiratory pathogen screening was conducted to detect HAdV and 17 other common respiratory pathogens, including influenza A virus (infA), influenza B virus (infB), respiratory syncytial virus (RSV), parainfluenza virus types 1–4 (PIV1–4), human metapneumovirus (HMPV), human rhinovirus (HRV), enterovirus (EV), four types of coronaviruses (HCoV-229E, -OC43, -NL63, and -HKU1), human bocavirus (HBoV), MP, and CP simultaneously using TaqMan real-time quantitative polymerase chain reaction (qPCR), as previously reported (Liu et al., 2013). Briefly, qPCR and RNA/DNA extraction kits were purchased from GuangzhouHuYanSuo Medical Technology Co., Ltd. RNA/DNA was extracted from 200 μL samples, according to the manufacturer’s protocol. The cycling conditions were 48°C for 5 min, 94°C for 2 min, and then 40 cycles of 94°C for 10 s and 55°C for 35 s. The amplified products were detected using the Applied Biosystems 7500 Real-Time PCR System (Life Technologies, Singapore). The sensitivity of the detection kits was 500 and 1000 copies/mL for the target DNA and RNA, respectively.

### Human Adenovirus Type 21 Identification

According to our previous study and the literature, HAdV-3 and -7 were the major types reported in children with acute respiratory disease (ARD) in Guangzhou, China, and HAdV-14, -21, -55, -C5, and -E4 were also reported but relatively rare. Therefore, HAdV-positive samples were subjected to further molecular typing for HAdV-3, -7, -14, -21, -55, -C5, and -E4 using TaqMan qPCR. The specific primers designed in-house, which probed the hexon or fiber genes of the different HAdV types, are shown in Supplementary Table 1. Probe qPCR Mix (TaKaRa, Dalian, China) was used according to the manufacturer’s protocol. Clinical characteristics, treatments, and outcomes of the HAdV-21-positive patients were collected retrospectively.

### Cells, Human Adenovirus Stocks, and Human Adenovirus Type 21-Positive Sample Culture

A549cells (ATCC, CCL-185) were cultured in Dulbecco’s minimum essential medium (DMEM) (Gibco, Grand Island, NY, United States) supplemented with 10% (v/v) fetal bovine serum (FBS) and 100 U/mL penicillin-streptomycin (Gibco, Grand Island, NY, United States) at 37°C and 5% (v/v) CO_2_. HAdV-21-positive samples were cultured in the A549cells at 37°C and 5% CO_2_ and maintained under standard conditions in DMEM supplemented with 2% (v/v) FBS and 100 U/mL penicillin-streptomycin. Inoculated cells were monitored daily for the cytopathic effect (CPE) and were harvested at almost full CPE. HAdV-3-Guangzhou01 (accession no. DQ099432), HAdV-7-CQ1198 (accession no. JX625134), and HAdV-21 reference stain AV-1645 (ATCC, accession no. AY601633) were used simultaneously for analysis of the cyto-pathogenicity of the HAdV-21 isolates. HAdV-3-Guangzhou01 and HAdV-7-CQ1198, from the State Key Laboratory of Respiratory Diseases, were collected from patients with severe pneumonia in Guangzhou in 2005 (Zhang et al., 2006) and Chongqing in 2010 (Chen et al., 2019), respectively. HAdV-21-AV-1645, which was first isolated in 1956 from a 1-year-old child with trachoma and conjunctivitis in Saudi Arabia (Bell et al., 1959), was kindly provided by Prof. Chenyang Li (Hexin Scientific, Guangzhou, China).

### Human Adenovirus Type 21 Genome Sequencing and Annotation

HAdV-21-positive samples were cultured and harvested. Viral genomic DNA was extracted using a TaKaRa Mini BEST Viral RNA/DNA Extraction Kit Ver.5.0 (TaKaRa) according to the manufacturer’s instructions. Next-generation sequencing was conducted with Illumina NovaSeq 6000 sequencer following a protocol from Synbio-Technologies (paired-end, 2 × 150 bp). The complete genome of HAdV-21 was assembled using CLC Genomics Workbench 11.0 (Qiagen, Redwood City, CA, United States). The complete genomes of the HAdV-21 isolates were annotated based on the annotation of HAdV-21 strain BB/201903 (accession no. MN686206) (Ye et al., 2020). Complete genome sequences were logged in the GenBank database.

### Phylogenetic Analysis and Human Adenovirus Sequences Used

Phylogenetic analysis was performed using Molecular Evolutionary Genetics Analysis (MEGA) version 11.0.8 (Tamura et al., 2011). Phylogenetic trees were constructed by the Neighbor-joining (NJ) method with 1,000 bootstrap replicates and default settings for all other parameters. HAdV sequences of the penton base, hexon, and fiber genes, and the genomes for phylogenetic analyses retrieved from GenBank are summarized in Supplementary Table 2. CLUSTALX was used for multiple sequence alignments of adenovirus proteins using default parameters.

### Viral Plaque Formation Assay

A549 cells were seeded into 6-well culture plates and incubated overnight to form dense monolayers with more than 90% confluence. After removal of the growth media, the cultures were inoculated with 0.4 mL of 10-fold serial dilutions of the viral stocks and incubated for 1 h at 37°C with rocking every 15 min. The viral inocula were removed by aspiration and 3 mL DMEM-agarose mulch [2% SeaPlaque GTG-agarose (Lonza) mixed 1:1 with 2 × DMEM medium containing 4% FBS] was added to each well. The agarose was allowed to solidify at room temperature (20–26°C). Plaque plates were incubated at 37°C and 5% CO_2_ for a total of 13 days, with 1.5 mL/well of DMEM-agarose mulch supplementation at 4- and 8-days post-infection. The plates were stained with 2 mL/well 20% ethanol, 2% paraformaldehyde, and 1% crystal violet overnight at room temperature. The diameters of the plaques were measured with the assistance of the VisionWorks software package.

### Human Adenovirus Growth Characteristics

To detect viral replication, the infected cells and the culture medium were harvested at 2, 12, 24, 36, 48, 96, and 120 h post-infection (h.p.i). The viral genomic DNA was extracted with a TaKaRa MiniBEST Viral RNA/DNA Extraction Kit Ver. 5.0 (TaKaRa, Dalian, China) according to the manufacturer’s instructions, and the viral genomic DNA copies were determined by quantitative PCR (qPCR) using previously described method.

### Neutralization Assays

Viruses completely inactivated by β-propiolactone (BPL; final concentration of 1:2000) were intraperitoneally injected into female BALB/c mice (4–6 weeks of age) at a concentration of 1010 genome copies/mouse, followed by two additional booster injections at 2-week intervals. Control mice were injected with phosphate-buffered saline (PBS). Two weeks after the final immunization, sera were collected, heat-inactivated and kept frozen for serology tests. All animal experiments were carried out in strict accordance with the guidelines of Guangdong Regulation for Administration of Laboratory Animals (2010) and approved by the Animal Ethic Committee of the First Affiliated Hospital of Guangzhou Medical University.

For *in vitro* neutralization experiments, antiserum from mice were serially diluted 2-fold in DMEM. 50 μL aliquots of each dilution was mixed with 50 μL of adenoviruses (100 TCID50). The antibod-virus mixtures were incubated for 1 h at 37°C and then transferred to 96-well plates containing 60–80% confluent monolayers of A549 cells. Monolayers were cultured in DMEM with 2% FBS for 4 days. The neutralization titer was determined as the maximum dilution of antiserum that completely inhibited viral growth as evidenced by the lack of cytopathic effect.

### Statistical Analysis

Statistical analyses between groups were performed using GraphPad Prism version 5 (GraphPad Software, lnc., San Diego, CA, United States). Differences between groups were calculated using the ANOVA and *t*-test. A *p*-value < 0.05 was considered statistically significant.

## Results

### Case Report of Human Adenovirus Type 21 Infection Among Pediatric Patients With Acute Respiratory Disease

**FIGURE 1:**
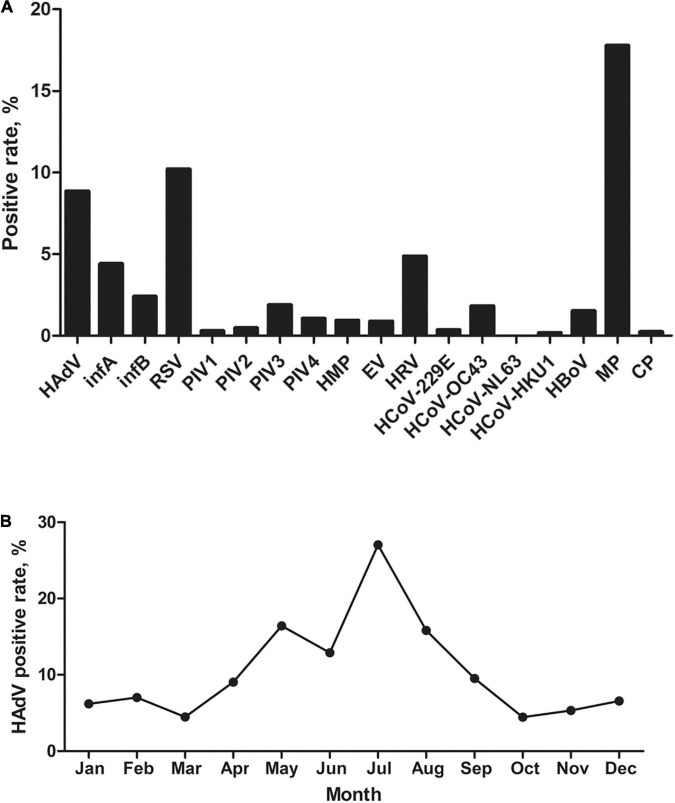
Detection of human adenovirus infection among 1,704 pediatric patients hospitalized with acute respiratory illness in Guangzhou, China in 2019. **(A)** Distribution of 18 respiratory pathogens in the children with ARD. **(B)** Monthly positive rate of HAdV infection in the hospitalized children.

In this study, 1704 hospitalized pediatric patients with ARD were enrolled between January and December 2019, with 845 (49.6%) patients infected with one or more of the 18 pathogens. The most frequently detected pathogens were MP (17.8%, 303/1,704), RSV (10.2%, 174/1,704), and HAdV (8.9%, 151/1,704). The positivity rates of the remaining fifteen pathogens were lower than 5.0% (Figure 1A). HAdV infection occurred year-round, mainly from May to August, and the incidence reached the peak in July (27.0%, 66/244) and the trough in October (4.4%, 7/158) (Figure 1B). Of the 151 HAdV-positive patients, two (1.3%) patients tested positive for HAdV-21 in June 2019 and September 2019. In the other HAdV-positive 149 patients, HAdV-3 (47.0%, 71/151), HAdV-7 (46.4%, 70/151), HAdV-4 (4.0%, 6/151), and HAdV-55 (1.3%, 2/151) were detected. In these two patients who are HAdV-21 positive there was not any other 17 pathogens and other HAdV types detected.

Data on the clinical characteristics, treatments, and outcomes of the two HAdV-21-positive patients were collected (Table 1). Both patients had similar initial symptoms of fatigue at the onset of the disease, and both were diagnosed with severe lower respiratory illness (SLRI) by radiologic testing. Patient 201906109 was diagnosed with pneumonia and sepsis; patient 201909107 was diagnosed with severe pneumonia. Patient 201906109 had sepsis, although no microbes were found by blood culture. The indexes of white cell count, procalcitonin (PCT), C-reactive protein (CRP), aspartate aminotransferase (AST), and D-dimer exceeded the upper limits of the normal ranges for both patients. Levels of creatine kinase (CK) and lactate dehydrogenase (LDH) were abnormal and differed between the two patients. While the patients had similar disease durations, patient 201909107, who had severe pneumonia in both lungs, was hospitalized for longer (9 days) than patient 201906109 (5 days) (Table 1).

**TABLE 1:** Clinical characteristics, treatments, and outcomes of the two patients infected with HAdV-21.

Characteristic	HAdV-21-positive patient	
	GZ06109	GZ09107
**Diagnosis of physician**	Pneumonia and sepsis	Severe pneumonia
**Radiologic findings**	Pneumonia	Pneumonia on both lungs
**Clinical characteristic**		
Gender	Male	Female
Age, year	4.6	1
Existing chronic disease	Rhinitis	Negative
Initial symptom	Repeated coughing and fever	Repeated coughing and fever
The highest temperature, °C	40.5	40.2
Sputum production	Yes	Yes
Shortness of breath	No	Yes
Fatigue	Yes	Yes
**Laboratory findings**		
The blood oxygen saturation under inhalation, %	99	88–90
Bacteria or fungus culture	Negative	Negative
White-cell count, ×10^9^/L	15.3, ↑	10.3, ↑
Lymphocyte count, ×10^9^/L	3.6	3.4
Platelet count, ×10^9^/L	342	332
Hemoglobin, g/L	101	104
Procalcitonin, ng/mL	1.47, ↑	0.2, ↑
Alanine aminotransferase, U/L	15	12
C-reactive protein, mg/dL	11.57, ↑	1.02, ↑
Aspartate aminotransferase, U/L	51.1, ↑	53, ↑
Creatine kinase, U/L	1152, ↑	86
Lactate dehydrogenase, U/L	215	642, ↑
D-dimer, ng/mL	509, ↑	1268, ↑
**Treatments**		
Symptomatic treatment	Anti-infection, anti-inflammation, intravenous fluid therapy, atomization inhalation treatment	Anti-infection, anti-inflammation, intravenous fluid therapy
Intravenous immune globulin	No	Yes
Mechanical ventilation	No	Yes
**Clinical outcomes- recovery duration**		
Total disease duration, day	20	19
Length of hospital stay, day	5	9

*Normal index range of test items: white cell count, 4–10 × 10^9^/L; lymphocyte count, 0.9–5.2 × 10^9^/L; platelet count, 100–400 × 10^9^/L; hemoglobin, 120–150 g/L; procalcitonin, 0–0.05 ng/mL; alanine aminotransferase, 5–40 U/L; C-reactive protein, 0–0.6 mg/dL; aspartate aminotransferase, 5–40 U/L; creatine kinase, 10–190 U/L; lactate dehydrogenase, 109–255 U/L; D-dimer, 68–494 ng/mL; “↑,” exceeding the upper limit of the normal range.*

### Genome Features and Phylogenetic Analysis of Human Adenovirus Type 21 Strains

**FIGURE 2:**
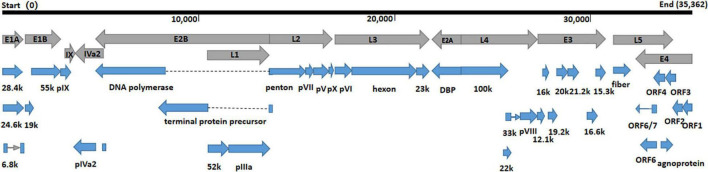
Transcriptional map and genome organization of HAdV-21 strain GZ09107. The genome is indicated by the black horizontal line marked at 10,000 bp intervals. The transcription units are designated by gray arrows, while blue arrows designate coding regions. Arrows reflect the transcriptional orientation of the coding transcripts.

**FIGURE 3:**
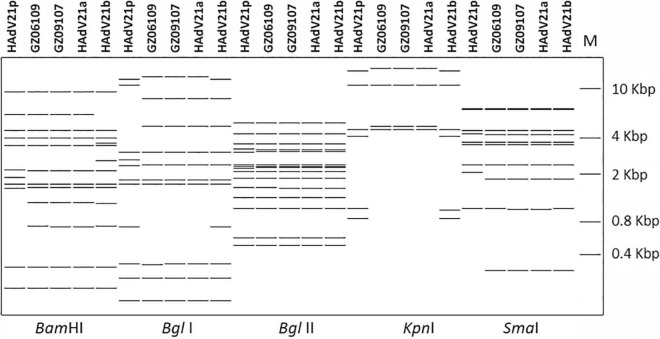
*In silico* restriction enzyme analysis (REA) of the HAdV-21 GZ06109 and GZ09107 sequences using the enzymes *BamH*I, *Bgl*I, *Bgl*II, *Kpn*I, and *Sma*I. Comparison was made with the HAdV-21a NHRC-5, HAdV-21p AV-1645, and HAdV-21b OHT-006. Genome type designations are based on unique arrays of restriction profiles with a panel of 6 endonucleases.

The whole genomes of HAdV-21 isolates GZ06109 and GZ09107 which was isolated from patient 201906109 and patient 201909107, respectively, were sequenced, annotated, and uploaded to the GenBank database with accession numbers MW091531 and MW151243, respectively. The identified genomes were 35,362 and 35,365 bp in length for GZ06109 and GZ09107, respectively. Figure 2 presents the genomic organization and transcription map for strain GZ09107 which was similar with that for strain GZ06109 and previously reported HAdV-21 NHRC5. The genome of GZ09107 was composed of 25.13% A, 23.66% T, 25.49% G, and 25.72% C, with a GC content of 51.21%, which was similar to other B1 subspecies (mean of 51%) (Seto et al., 2010). The two isolates GZ06109 and GZ09107 were identified as HAdV-21a based on restriction fragment length polymorphism (RFLP) analysis of viral genomes (Figure 3).

**FIGURE 4:**
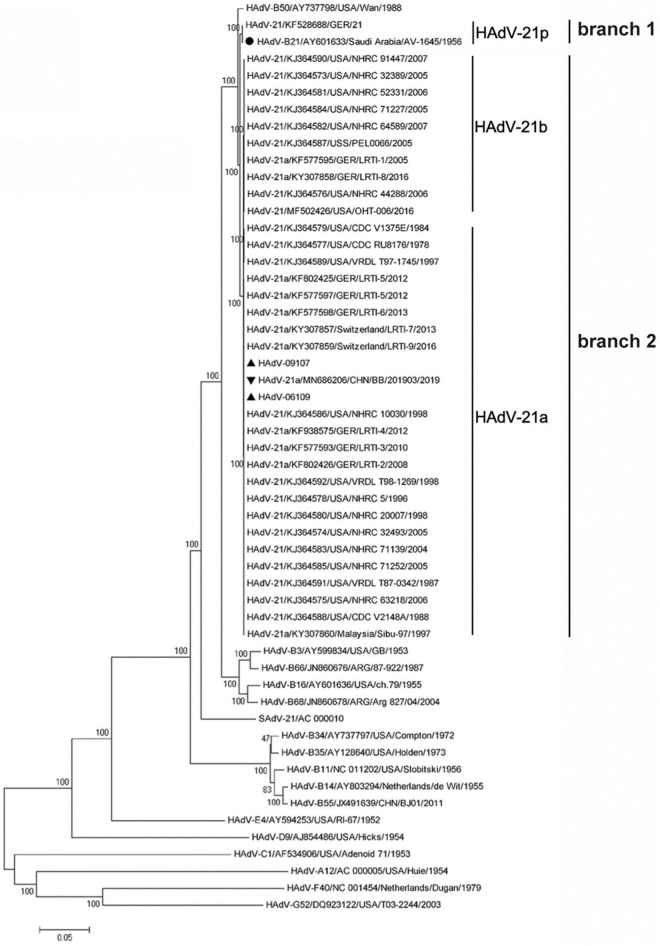
Phylogenetic analysis of HAdV-21 strains GZ06109 and GZ09107. The nucleotide sequences of the whole genome of the HAdV strains were analyzed for their phylogenetic relationships using the Neighbor-Joining method with 1,000 bootstrap replicates implemented in the MEGA 11.0.8 software package. For reference, taxon names include the genome type, corresponding GenBank accession number, country of isolation, strain name, and year of isolation. The two HAdV-21 strains isolated in this study are marked with “▲”; “▼”, strain isolated from Bangbu (BB/201903), China in 2019; “🌑,” reference standard HAdV-21 isolated in Saudi Arabia in 1945.

Phylogenetic relationships were inferred based on available sequences of HAdV-21 genomes and selected proteins. Whole genome analysis showed the two HAdV-21 isolates in this study were tightly clustered in one lineage with strains of subtype HAdV-21a (Figure 4). The only two HAdV-21 strains of subtype HAdV-21p, the prototype AV-1645 strain and the GER strain, were clustered in one branch (branch 1). All other HAdV-21 strains were clustered in another branch (branch 2) which could be subdivided into two lineages, subtype HAdV-21a and HAdV-21b. The percent similarity of whole genomes was 99.79–99.99% within a branch and 98.82–99.00% between two branches. The phylogenetic trees based on the structural proteins, hexon, penton base and fiber also revealed the two HAdV-21 isolates clustered with strains from subtype HAdV-21a (Supplementary Figure 1). In addition, the phylogenetic relationship between HAdV-50 and HAdV-21 was closer than with other types (Figure 4). HAdV-50 was clustered with HAdV-21 strains in the phylogenetic trees based on whole genomes, penton base and fiber, but not in the hexon phylogenetic tree (Supplementary Figure 1).

The percent identities of the whole genomes of the two HAdV-21 isolates with the most closely related strain, HAdV-21-BB/201903 of subtype 21a, were both 99.97%; and with the prototype AV-1645 strain of subtype 21p were 98.86%; with the subtype 21b strain OHT-006 were 99.81% (Table 2). The percent similarity of fiber gene of three strains GZ06109, GZ09107, and BB/201903 from subtype 21a, and OHT-006 from subtype 21b were 100%. We also did comparative genomic analysis of HAdV-21a strains GZ06109, GZ09107, and BB with the reference HAdV-21a strain NHRC 5 (Table 3). Most mutations happened in the non-coding regions. The non-synonymous mutations happened in large T antigen, hypothetical 12.6 kDa protein of E2B gene, penton base, or agnoprotein of E4 gene. Compared with GZ09107, GZ06109 contained one amino acid deletion mutation in the penton base (GCG, alanine) and one amino acid non-synonymous mutation in large T antigen (H → Y) (Table 3).

**TABLE 2:** Percent identities of genomes and capsid protein gene sequences of the isolated strains GZ06109 and GZ09107 with representative HAdV-21 strains.

Strains	GZ06109				GZ09107			
	Genome	Hexon	Penton base	Fiber	Genome	Hexon	Penton base	Fiber
BB/201903^a^	99.97	100	99.82	100	99.97	100	100	100
OHT-006^b^	99.81	99.79	99.64	100	99.81	99.79	99.82	100
AV-1645^c^	98.86	99.16	99.26	99.38	98.86	99.16	99.47	99.38

*^a^The most closely related strain of subtype HAdV-21a, HAdV-21a/MN686206/CHN/BB/201903/2019, which was also detected in China in 2019.*

*^b^A strain of subtype HAdV-21b, HAdV-21/MF502426/United States/OHT-006/2016, isolated in United States in 2016.*

*^c^The HAdV-21 prototype strain of subtype 21p, HAdV-B21/AY601633/Saudi Arabia/AV-1645/1956.*

**TABLE 3:** Comparative genomic analysis of HAdV-21a strains GZ06109, GZ09107, and BB with the reference HAdV-21a strain NHRC 5.

Region	Gene	Position	Mutation in DNA			Mutation in AA (dN)		
			GZ06109	GZ09107	CHN/BB	GZ06109	GZ09107	CHN/BB
5′-UTR	NCR	164	C → G	C → G	C → G	–	–	–
	NCR	256	▼T	▼T	▼T	–	–	–
	NCR	266	C → T	C → T	C → T	–	–	–
E1B	Small T antigen	1950	–	C → T	–	–	dS	–
	Large T antigen	1950	–	C → T	–	–	**H → Y**	–
	pIX	3785	G → C	G → C	G → C	dS	dS	dS
E2B	Hypothetical 12.6 kDa protein	8271	G → T	G → T	G → T	**P → T**	**P → T**	**P → T**
L1	NCR	10577	▲T	▲T	▲T	–	–	–
	NCR	10836	▼TTTG	▼TTTG	▼TG	–	–	–
	NCR	10838	G → T	G → T	G → T	–	–	–
L2	Penton base	13980	▲GCG	–	–	**▲A**	–	–
	NCR	15542	▼AAAA	▼AAAA	▼AAAAAAAAAA	–	–	–
E2A	E2A	23079	A → G	A → G	A → G	dS	dS	dS
L4	100 kDa protein	25040	A → C	A → C	A → C	dS	dS	dS
E3	20 kDa protein	28764	–	G → A	–	–	dS	–
	NCR	29207	▼T	▼T	▼T	–	–	–
	NCR	29788	▼T	▼T	▼T	–	–	–
	NCR	30092	▼TT	▼TT	▼TT	–	–	–
	NCR	31216	▼A	▼A	▼A	–	–	–
L5	NCR	32374	▼A	▼A	▼A	–	–	–
E4	Agnoprotein	34062	G → T	G → T	G → T	**L → M**	**L → M**	**L → M**
3′-UTR	NCR	35116	▼AA	▼AA	▼AA	–	–	–

*Differences in nucleic acid and amino acid sequences are shown along with their genomic locations and coding consequences.*

*The reference genome is HAdV-21a strain NHRC 5.*

*NCR, non-coding region; ▼, insertion; ▲, deletion; –, no change or not applicable; dS, synonymous; dN, non-synonymous.*

*The bold values show amino acids of non-synonymous mutation.*

Multiple sequence alignment was done using the full proteins, hexon, penton base or fiber from HAdV-21 subtypes 21a, 21b, 21p, and selected HAdV types according to the phylogenetic trees. Compared with HAdV-21p, HAdV-21a contained some amino acid mutations in the hexon (Supplementary Figure 2A). As expected, most mutations happened in seven HVRs, and there was no change between HAdV-21a and HAdV-21b in branch 2 (Supplementary Figure 2A). Strikingly, compared with HAdV-21p, HAdV-21a and HAdV-21b contained a 15 amino acid peptide deletion mutation, _313_TEAAKAAAIAKANIV_327_, and a 2 amino acid insertion mutation, _348_ET_349_, in the penton base (Supplementary Figure 2B). However, HAdV-50 and HAdV-35 penton bases have the 15 amino acid peptide insertion, and the 2 amino acid deletion, in line with HAdV-21p (Supplementary Figure 2B). In fiber protein, there was no change between HAdV-21a and HAdV-21b in branch 2. Compared with HAdV-21p, HAdV-21a and HAdV-21b contained three amino acid mutations, _49_N → K, _70_I → T, _279_R → H. Compared with HAdV-21p, HAdV-50 contained only one amino acid mutation, _84_A → V (Supplementary Figure 2C).

### Growth Characteristics of Human Adenovirus Type 21 Strains *in vitro*

**FIGURE 5:**
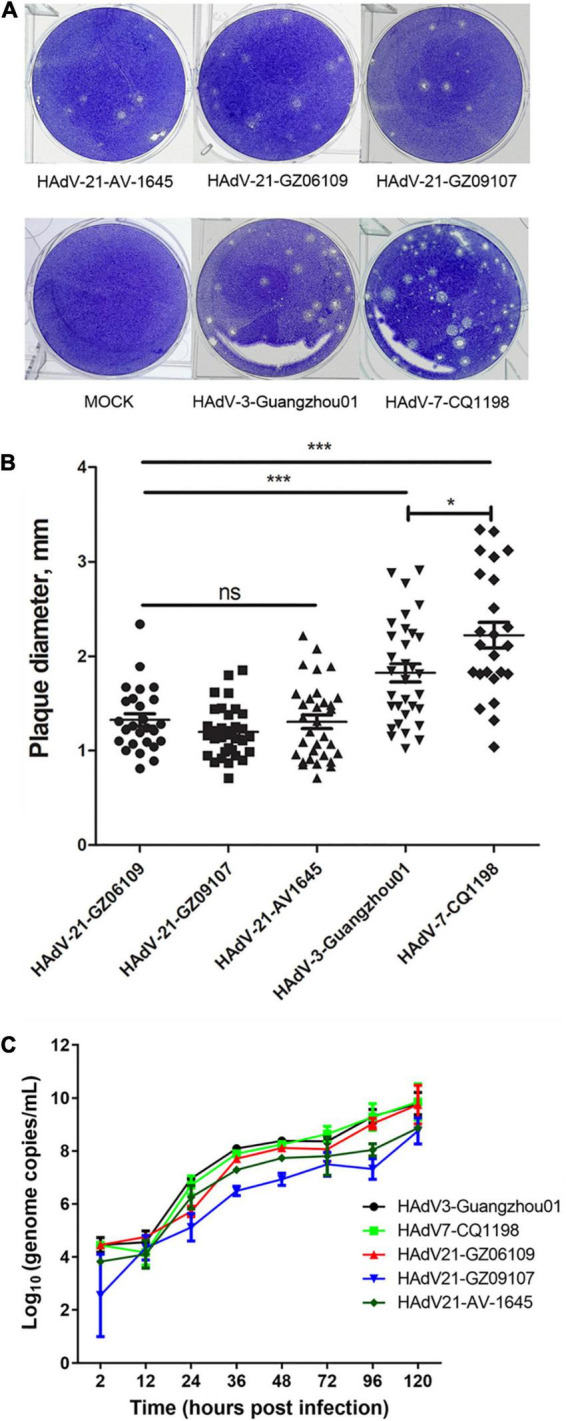
Plaque formation and size distribution of the HAdV-21 isolates and references HAdV-21-AV-1645, HAdV-3-Guangzhou01, and HAdV-7-CQ1198. **(A)** Plaque formation. **(B)** Size distribution. Plaque plates were incubated and stained with crystal violet for a total of 13 days in 6-well culture plates. ns, not significant; *, *p* < 0.05; ***, *p* < 0.001. **(C)** Growth curves of HAdV strains. Virus genome copies were quantified by Q-PCR after different hours post-infection of HAdVs in A549 cells.

Human adenovirus type 21 strains GZ06109 and GZ09107 were successfully isolated from the clinical samples testing positive for HAdV-21 and cultured. Cyto-pathogenicity analyses of HAdV-21 strains GZ06109, GZ09107, AV-1645, HAdV-3-Guangzhou01, and HAdV-7-CQ1198 were performed using the plaque formation assay. Plaques formed by the HAdV-21 strains were smaller and had more vague boundaries than those from HAdV-7-CQ1198 and HAdV-3-Guangzhou01 (Figure 5A). Plaque sizes were significantly different among the three HAdV types (*p* < 0.05), but not among the three HAdV-21 strains (Figure 5B). Two days after infection with the HAdV-21 strains at a multiplicity of infection (MOI) of 2 PFU/cell, the A549 cells showed similar typical CPE. The HAdV-21 strains had similarly high titers (approximately 10^–9^ PFU/mL). The replication of the strains was compared through genome quantification using Q-PCR (Figure 5C). The replication kinetics of HAdV-21 strain GZ06109 and GZ09107 were identical to that of HAdV-21 strain AV-1645.

### Antigenic Variability Among Subtypes of Human Adenovirus Type 21

**FIGURE 6:**
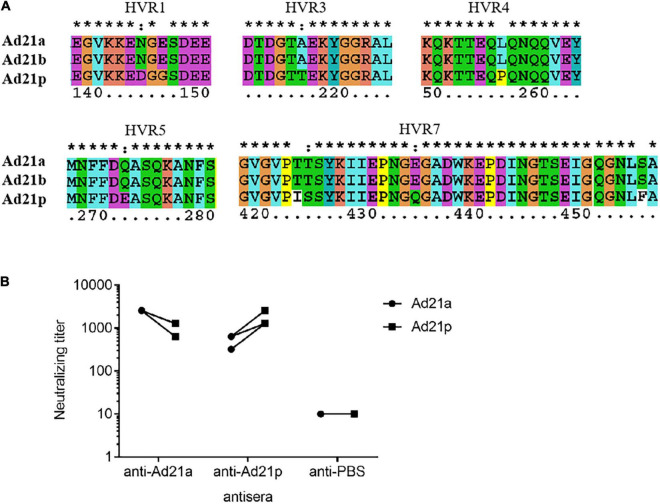
Antigenic variability among HAdV-21 strains. **(A)** Multiple sequence alignment of several hexon proteins from subtypes HAdV-21a (GZ06109), 21b (OHT-006), and 21p (AV-1645). Only five hypervariable regions with substitution are shown. The number shows the position of amino acid in the hexon protein. *, conserved amino acid; ∙, either size or hydropathy is conserved; and:, both size and hydropathy are conserved. Gaps used to optimize alignments are indicated by *dashes.* **(B)** *In vitro* micro-neutralization of HAdV-21 with hyperimmune sera from mice. Antisera were collected from mice immunized with purified HAdV-21a (GZ06109) and 21p (AV-1645) (*n* = 4). Cross-neutralization tests against these two strains were performed. The anti-PBS sera were used as the negative control.

Hexon and fiber are the two major neutralization antigens of adenovirus. The fiber proteins were highly conserved in all HAdV-21 strains with a variation of only one or two amino acids. Detailed phylogenetic analysis for hexon also indicated the existence of the two branches (Supplementary Figure 1). The phylogenetic tree based on the partial hexon amino acid sequences containing all HVRs available in GenBank showed two branches with more distinct clusters (data not shown). Branch 1 include strains of subtype 21p; branch 2 include strains of subtype 21a and 21b. Multiple sequence alignments revealed changes between the two branches in HVR1, HVR3, HVR4, HVR5, and HVR7. The two HAdV-21 branches showed no variation in HVR2 and HVR6 (Supplementary Figure 2A). Among strains of the same branch, there was no amino acid substitution (Figure 6A). Multiple alignments also revealed the mutations between the two branches in fiber protein (Supplementary Figure 2C).

The HAdV-21 GZ06109 and GZ09107 strains were subtype 21a; HAdV-21 prototype AV-1645 strain was subtype 21p. As shown in Figure 6B, the hyperimmune antisera were specific for the corresponding virus with titers ranging from 1,280 to 2,560. Serum from mice hyperimmunized with HAdV-21a GZ06109 (branch 2) yielded a 2–4-fold lower titer to HAdV-21p AV-1645 (branch 1) compared to that for HAdV-21a GZ06109. Serum from mice hyperimmunized mice with HAdV-21p AV-1645 yielded a 2–4-fold lower titer to HAdV-21a GZ06109 than to HAdV-21p AV-1645.

## Discussion

Among the more than 100 types and seven species (A–G) of HAdV, species B is of particular importance in ARI.^1^ Although HAdV-21 is an important member of HAdV species B, our understanding of this type is inadequate. In this study, we analyzed the epidemiologic characteristics, genome features, and cyto-pathogenicity of HAdV-21 infection in Guangzhou, southern China. Two HAdV-21 strains were isolated and characterized *in vitro*. The antigenic variability among subtypes of human adenovirus 21 was further analyzed.

Of the 1,704 participants in this study, 8.9% of the patients were infected with HAdV, making it the third most prevalent pathogen detected (Figure 1). This positive rate was higher than that reported for previous years in this region (5%, 213/4242) (Liu et al., 2014). The main HAdV types detected were type 7 and 3, which is largely consistent with previous reports (Liu et al., 2014; Lin et al., 2017; Duan et al., 2021). Only two sporadic cases of HAdV-21, GZ06109 and GZ09107, were identified in June and September. The low prevalence of HAdV-21 may signify low immunity against this type in the general population, increasing its potential to cause an epidemic.

Both HAdV-21-positive patients presented with severe LRI (Table 1), which highlights the need for increased awareness of HAdV-21 infections. The HAdV-21 infections had similar initial symptoms at onset of illness, and multiple indexes exceeded the normal ranges, such as white cell count, PCT, CRP, AST, and D-Dimer. Of them, the abnormal AST is an indicator of hepatitis or liver injury. These factors may help physicians judge and treat for infection (Table 1), although definitive diagnosis will require laboratory screening as many respiratory viruses have similar manifestations. Because there are no specific treatments against HAdVs, only symptomatic treatment can be used at present. Previous studies have shown that HAdV-21 can cause nosocomial infections in immune-compromised patients (Philo et al., 2018), highlighting the importance of prevention and control of this type.

To improve our understanding of HAdV-21, we cultured the two HAdV-21 isolates, GZ06109 and GZ09107. Sequencing and annotation of their genomes revealed similar structures to other members of HAdV species B (Cheng et al., 2018; Figure 2). The HAdV-21 isolates’ genomes constituted a clade with HAdV-21a (Kajon et al., 2015; Figures 3, 4). The two HAdV-21 strains in this study had highest genome identity (99.97%) with strain HAdV-21-BB/201903 (accession no. MN686206), which was the first isolate in China (Ye et al., 2020; Table 2). This suggests that HAdV-21 isolates prevalent in China have a high degree of kinship and are from the same source, although there is insufficient data to identify the potential source.

To analyze the cyto-pathogenicity of HAdV-21, plaque formation assays were conducted (Figure 5). The plaques formed by the three HAdV-21 strains showed similar characteristics (Figure 5), while they were significantly smaller than plaques from HAdV-7 and HAdV-3 (*p* < 0.001), and had poorly defined edges. It was also found that plaques formed by HAdV-7 were significantly larger than those of HAdV-3 (*p* < 0.05), which was consistent with the previous report (Fu et al., 2019). The plaque features suggest that the virulence and infectivity of three types *in vitro* are HAdV-21 < HAdV-3 < HAdV-7, but at the same time, we should also note that the patients with HAdV-21 infection in this study are all severe LRI-related, indicating that the pathogenic factors of HAdV-21 are diverse. There are too few reports on HAdV-21 to determine its overall pathogenic characteristics; thus, more research on this type is urgently needed.

Seven genotypes of HAdV-21 have been identified based on RFLP analysis of viral genomes (Kajon et al., 2015). Determining antigenic profiles is crucial for characterizing epidemic strains and assessing vaccine efficacy, while neutralization profiles and epitopes are not indicated by RFLP results. Hexon and fiber are the major antigens of HAdV. Most serotype-specific neutralizing epitopes of HAdV are located in multiple HVRs within the hexon protein. Phylogenetic analysis of HAdV-21 genomes showed that these strains could be divided into two branches (Figure 4). Our analyses for hexon also confirmed the two branches were genetically distinct (Supplementary Figure 1). Multiple sequence alignment of HAdV-21 hexon proteins indicated that HVR1, 4, and 7 are conserved within the same branch, but were altered between the two branches, possibly contributing to the drift of neutralization profiles (Supplementary Figure 2A and Figure 6A). Cross-neutralization test results demonstrated the two branches were dramatically distinct (Figure 6B). Although the antisera of the two branches could neutralize each other, the neutralization titer was reduced 2–4-fold. Strains of subtypes HAdV-21a and 21b of branch 2 have predominated since the 1960s. It is important to note that the neutralization epitopes of branch 2 strains remain virtually unchanged. We believe our findings and the two isolated HAdV-21a strains could be helpful in novel HAdV-21 vaccine and drug development.

The main limitation of this study is that selection bias may have occurred, because the sample comes from two patients, and there is a lack of samples from outpatient clinics and healthy people. Secondly, the number of HAdV-21-positive cases is still small. This may lead to deviations in the understanding of HAdV-21 infection, especially the epidemiological characteristics.

## Conclusion

Human adenovirus type 21 infections of children were firstly reported in China. HAdV-21 infection was sporadic in children in Guangzhou, southern China. However, HAdV-21 infection still needs sufficient attention because it can cause severe LRI. Two HAdV-21a isolates showed a high degree of similarity in genome sequence and growth characterization. We found HAdV-21 could be classified into two branches using genome sequence and antigenic analysis. Mutations in five HVRs between the two branches alter antigenic profiles. Our results are important for understanding the HAdV-21 epidemiology and pathogenic, and for developing HAdV-21 vaccine.

## Data Availability Statement

The original contributions presented in this study are included in this article/Supplementary Material, further inquiries can be directed to the corresponding authors.

## Ethics Statement

The studies involving human participants were reviewed and approved by the Ethics Committee of First Affiliated Hospital of Guangzhou Medical University. Written informed consent to participate in this study was provided by the participants’ legal guardian/next of kin. The animal study was reviewed and approved by the Animal Ethics Committee of First Affiliated Hospital of Guangzhou Medical University.

## Author Contributions

WL, QL, XT, and RZ: conceptualization and funding acquisition. WL, LZ, YC, and XT: methodology. WL, LZ, YC, QL, XT, and RZ: formal analysis. WL, LZ, YC, QZ, SQ, YW, DX, SG, XL, and JD: investigation. DC and QL: resources. WL, XT, LZ, YC, and QL: writing – original draft preparation. XT and RZ: writing – review and editing. All authors have read and agreed on the final manuscript.

## Conflict of Interest

The authors declare that the research was conducted in the absence of any commercial or financial relationships that could be construed as a potential conflict of interest.

## Publisher’s Note

All claims expressed in this article are solely those of the authors and do not necessarily represent those of their affiliated organizations, or those of the publisher, the editors and the reviewers. Any product that may be evaluated in this article, or claim that may be made by its manufacturer, is not guaranteed or endorsed by the publisher.

## Footnotes

1
http://hadvwg.gmu.edu/

## References

[B1] Becroft D. M. (1971). Bronchiolitis obliterans, bronchiectasis, and other sequelae of adenovirus type 21 infection in young children. *J. Clin. Pathol.* 24 72–82. 10.1136/jcp.24.1.72 4324685 PMC478030

[B2] Bell S. D. Mc C. D. Murray E. S. Chang R. S. Snyder J. C. (1959). Adenoviruses isolated from Saudi Arabia. I. Epidemiologic features. *Am. J. Trop. Med. Hyg.* 8 492–500. 10.4269/ajtmh.1959.8.492 13670377

[B3] Chen S. Fan Y. Zhang L. Tian X. Zhou R. (2019). Preliminary Comparison of in vitro Infection Characteristics of Human Adenovirus Causing Acute Respiratory-tract Infection. *Chin. J. Virol.* 35 741–747.

[B4] Chen S. Y. Liu W. Xu Y. Qiu S. Chen Y. Tian X. (2020). Epidemiology and Genetic Variabilities of Human Adenovirus Type 55 Reveal Relative Genome Stability Across Time and Geographic Space in China. *Front. Microbiol.* 11:606195. 10.3389/fmicb.2020.606195 33343550 PMC7738467

[B5] Cheng Z. Yan Y. Jing S. Li W. G. Chen W. W. Zhang J. (2018). Comparative Genomic Analysis of Re-emergent Human Adenovirus Type 55 Pathogens Associated With Adult Severe Community-Acquired Pneumonia Reveals Conserved Genomes and Capsid Proteins. *Front. Microbiol.* 9:1180. 10.3389/fmicb.2018.01180 29922263 PMC5996824

[B6] Darougar S. Pearce R. Gibson J. A. McSwiggan D. A. (1978). Adenovirus type 21 keratoconjunctivitis. *Br. J. Ophthalmol.* 62 836–837. 10.1136/bjo.62.12.836 216386 PMC1043368

[B7] Deng J. Qian Y. Zhao L. Q. Zhu R. N. Wang F. Sun Y. (2007). [Identification and typing for adenovirus by multiplex nest-PCR]. *Zhonghua Liu Xing Bing Xue Za Zhi* 28 781–784. 18080566

[B8] Duan Y. Li C. Deng L. An S. Zhu Y. Wang W. (2021). Genetic Analysis of Human Adenovirus Type 7 Strains Circulating in Different Parts of China. *Virol. Sin.* 36 382–392. 10.1007/s12250-020-00334-y 33400092 PMC7783484

[B9] Fu Y. Tang Z. Ye Z. Mo S. Tian X. Ni K. (2019). Human adenovirus type 7 infection causes a more severe disease than type 3. *BMC Infect. Dis.* 19:36. 10.1186/s12879-018-3651-2 30626350 PMC6327436

[B10] Hage E. Huzly D. Ganzenmueller T. Beck R. Schulz T. F. Heim A. A. (2014). human adenovirus species B subtype 21a associated with severe pneumonia. *J. Infect.* 69 490–499. 10.1016/j.jinf.2014.06.015 24975176

[B11] Henson D. Mufson M. A. (1971). Myocarditis and pneumonitis with type 21 adenovirus infection. Association with fatal myocarditis and pneumonitis. *Am. J. Dis. Child.* 121 334–336. 10.1001/archpedi.1971.02100150108015 4323839

[B12] Ismail A. M. Lee J. S. Lee J. Y. Singh G. Dyer D. W. Seto D. (2018). Adenoviromics: Mining the Human Adenovirus Species D Genome. *Front. Microbiol.* 9:2178. 10.3389/fmicb.2018.02178 30254627 PMC6141750

[B13] James A. G. Lang W. R. Liang A. Y. Mackay R. J. Morris M. C. Newman J. N. (1979). Adenovirus type 21 bronchopneumonia in infants and young children. *J. Pediatr.* 95 530–533. 10.1016/s0022-3476(79)80756-8 225460

[B14] Ji T. Li L. Li W. Zheng X. Ye X. Chen H. (2021). Emergence and characterization of a putative novel human adenovirus recombinant HAdV-C104 causing pneumonia in Southern China. *Virus Evol.* 7:veab018. 10.1093/ve/veab018 33732504 PMC7953211

[B15] Kajon A. E. Hang J. Hawksworth A. Metzgar D. Hage E. Hansen C. J. (2015). Molecular Epidemiology of Adenovirus Type 21 Respiratory Strains Isolated From US Military Trainees (1996-2014). *J. Infect. Dis.* 212 871–880. 10.1093/infdis/jiv141 25748322

[B16] Lang W. R. Howden C. W. Laws J. Burton J. F. (1969). Bronchopneumonia with serious sequelae in children with evidence of adenovirus type 21 infection. *Br. Med. J.* 1 73–79. 10.1136/bmj.1.5636.73 4302627 PMC1981971

[B17] Lee C. S. Lee A. Y. Akileswaran L. Stroman D. Najafi-Tagol K. Kleiboeker S. (2018). Determinants of Outcomes of Adenoviral Keratoconjunctivitis. *Ophthalmology* 125 1344–1353. 10.1016/j.ophtha.2018.02.016 29602567 PMC6109430

[B18] Lin M. R. Yang S. L. Gong Y. N. Kuo C. C. Chiu C. H. Chen C. J. (2017). Clinical and molecular features of adenovirus type 2, 3, and 7 infections in children in an outbreak in Taiwan, 2011. *Clin. Microbiol. Infect.* 23 110–116. 10.1016/j.cmi.2016.11.004 27851998 PMC7129580

[B19] Liu W. K. Chen D. H. Liu Q. Liang H. X. Yang Z. F. Qin S. (2011). Detection of human bocavirus from children and adults with acute respiratory tract illness in Guangzhou, southern China. *BMC Infect. Dis.* 11:345. 10.1186/1471-2334-11-345 22168387 PMC3267697

[B20] Liu W. K. Liu Q. Chen D. H. Liang H. X. Chen X. K. Huang W. B. (2013). Epidemiology and clinical presentation of the four human parainfluenza virus types. *BMC Infect. Dis.* 13:28. 10.1186/1471-2334-13-28 23343342 PMC3560251

[B21] Liu W. K. Liu Q. Chen de H. Liang H. X. Chen X. K. Chen M. X. (2014). Epidemiology of acute respiratory infections in children in guangzhou: a three-year study. *PLoS One* 9:e96674. 10.1371/journal.pone.0096674 24797911 PMC4010508

[B22] Ooi M. H. Wong S. C. Clear D. Perera D. Krishnan S. Preston T. (2003). Adenovirus type 21-associated acute flaccid paralysis during an outbreak of hand-foot-and-mouth disease in Sarawak, Malaysia. *Clin. Infect. Dis.* 36 550–559. 10.1086/367648 12594634

[B23] Pereira M. S. (1963). Occurrence of Adenovirus Type 21 in Great Britain. *Br. Med. J.* 1 728–729. 10.1136/bmj.1.5332.728 20789699 PMC2123241

[B24] Pfortmueller C. A. Barbani M. T. Schefold J. C. Hage E. Heim A. Zimmerli S. (2019). Severe acute respiratory distress syndrome (ARDS) induced by human adenovirus B21: report on 2 cases and literature review. *J. Crit. Care* 51 99–104. 10.1016/j.jcrc.2019.02.019 30798099 PMC7172394

[B25] Philo S. E. Anderson B. D. Costa S. F. Henshaw N. Lewis S. S. Reynolds J. M. (2018). Adenovirus Type 21 Outbreak Among Lung Transplant Patients at a Large Tertiary Care Hospital. *Open Forum Infect. Dis.* 5:ofy188. 10.1093/ofid/ofy188 30151413 PMC6105090

[B26] Prusinkiewicz M. A. Mymryk J. S. (2019). Metabolic Reprogramming of the Host Cell by Human Adenovirus Infection. *Viruses* 11:141. 10.3390/v11020141 30744016 PMC6409786

[B27] Radke J. R. Cook J. L. (2018). Human adenovirus infections: update and consideration of mechanisms of viral persistence. *Curr. Opin. Infect. Dis.* 31 251–256. 10.1097/QCO.0000000000000451 29601326 PMC6367924

[B28] Sandkovsky U. Vargas L. Florescu D. F. (2014). Adenovirus: current epidemiology and emerging approaches to prevention and treatment. *Curr. Infect. Dis. Rep.* 16:416. 10.1007/s11908-014-0416-y 24908344

[B29] Seto J. Walsh M. P. Mahadevan P. Zhang Q. Seto D. (2010). Applying genomic and bioinformatic resources to human adenovirus genomes for use in vaccine development and for applications in vector development for gene delivery. *Viruses* 2, 1–26. 10.3390/v2010001 21994597 PMC3185558

[B30] Tamura K. Peterson D. Peterson N. Stecher G. Nei M. Kumar S. (2011). MEGA5: molecular evolutionary genetics analysis using maximum likelihood, evolutionary distance, and maximum parsimony methods. *Mol. Biol. Evol.* 28 2731–2739. 21546353 10.1093/molbev/msr121 PMC3203626

[B31] van der Avoort H. G. Adrian T. Wigand R. Wermenbol A. G. Zomerdijk T. P. de Jong J. C. (1986). Molecular epidemiology of adenovirus type 21 in the Netherlands and the Federal Republic of Germany from 1960 to 1985. *J. Clin. Microbiol.* 24 1084–1088. 10.1128/jcm.24.6.1084-1088.1986 3023438 PMC269103

[B32] van der Veen J. Oei K. G. Abarbanel M. F. (1969). Patterns of infections with adenovirus types 4, 7 and 21 in military recruits during a 9-year survey. *J. Hyg.* 67 255–268. 10.1017/s0022172400041668 4307899 PMC2130709

[B33] Wright J. Couchonnal G. Hodges G. R. (1979). Adenovirus type 21 infection. Occurrence with pneumonia, rhabdomyolysis, and myoglobinuria in an adult. *JAMA* 241 2420–2421. 10.1001/jama.241.22.2420 439320

[B34] Ye F. Han Y. Zhu J. Li P. Zhang Q. Lin Y. (2020). First Identification of Human Adenovirus Subtype 21a in China With MinION and Illumina Sequencers. *Front. Gen.* 11:285. 10.3389/fgene.2020.00285 32318094 PMC7155751

[B35] Zhang Q. Su X. Gong S. Zeng Q. Zhu B. Wu Z. (2006). Comparative genomic analysis of two strains of human adenovirus type 3 isolated from children with acute respiratory infection in southern China. *J. Gen. Virol.* 87 1531–1541. 10.1099/vir.0.81515-0 16690917

