# Supplementary material for: Human Adenovirus Subtype 21a Isolates From Children With Severe Lower Respiratory Illness in China
Source: Front Microbiol. 2022 Jun 16;13:924172. doi: 10.3389/fmicb.2022.924172 (PMC9244545; doi:10.3389/fmicb.2022.924172)
Supplement: Supplementary file 1 [file Table_1.DOCX]

**Supplemental Material**

**Table S1. Primers and probes for identification of different HAdV types**

| **HAdV Type** | **Primer and Probe** | **Sequence (5’-3’)** |
| --- | --- | --- |
| HAdV-21 | AD21F | GGGCTTGTTAATGGCTACGTATCT |
|  | AD21R | TGCTGACTTTTGTGTGAACATTTG |
|  | AD21Probe | FAM-AGTTGGTGTATCAGACACT-MGB |
| HAdV-3 | AD3F | CGAGACCTCCTACCCATGAACTAA |
|  | AD3R | GACTGTATTGCTGATTTCAAGTAAGTGTCT |
|  | AD3Probe | FAM- TCATTGCCCCTACCTTACCCAATCCAA-BHQ1 |
| HAdV-7 | AD7F | CAGGAGAAGAAAGAGCAGTAACTACCA |
|  | AD7R | TGCAGTAATGTCTTTCCCAATTTCTA |
|  | AD7Probe | FAM-CAAACACATTTGGCATTGCTTCCATGAA-BHQ1 |
| HAdV-14 | AD14F | GTGAAAGGCGGACAGGCAAA |
|  | AD14R | CAAAAAAGTTCATGTCAATGTCATATTCAA |
|  | AD14Probe | FAM-TGAAAACAGAAGAAGGCAACAA-BHQ1 |
| HAdV-55 | AD55F | AAGGCGGTCAGGCAAAACC |
|  | AD55R | CCATGTCAATATCATATTCGACTTTCTGA |
|  | AD55Probe | FAM-AAAACAACGGAGCAGCCA-BHQ1 |
| HAdV-5 | ADV5F | ACGATGACAACGAAGACGAAGTAG |
|  | ADV5R | GGCGCCTGCCCAAATAC |
|  | ADV5Probe | FAM-CGAGCAAGCTGAGCAGCAAAAAACTCA-BHQ1 |
| HAdV-4 | AD4F | GCAAGCTACTGCTCTTCCGAC |
|  | AD4R | GTGTGATGGCAGGGTCCCGAG |
|  | AD4Probe | FAM- CTTCCTACCAGGGATCTATCAGTGCGTC-BHQ1 |

**Table S2. Sequences of adenovirus species A–G used for phylogenetic analysis**

| **Type** | **Strain** | **Country** | **Year isolated** | **GenBank accession number** |
| --- | --- | --- | --- | --- |
| **HAdV-B21** | GZ06109^#^ | China | 2019 | MW091531 |
|  | GZ09107^#^ | China | 2019 | MW151243 |
|  | BB/201903 | China | 2019 | MN686206 |
|  | AV-1645 | Saudi Arabia | 1956 | AY601633 |
|  | OHT-006 | USA | 2016 | MF502426 |
|  | LRTI-1 | Germany | 2005 | KF577595 |
|  | LRTI-8 | Germany | 2016 | KY307858 |
|  | PEL0066 | USS | 2005 | KJ364587 |
|  | NHRC 64589 | USA | 2007 | KJ364582 |
|  | NHRC 71227 | USA | 2005 | KJ364584 |
|  | NHRC 52331 | USA | 2006 | KJ364581 |
|  | NHRC 44288 | USA | 2006 | KJ364576 |
|  | NHRC 32389 | USA | 2005 | KJ364573 |
|  | NHRC 91447 | USA | 2007 | KJ364590 |
|  | LRTI-3 | Germany | 2010 | KF577593 |
|  | LRTI-5 | Germany | 2012 | KF802425 |
|  | LRTI-5 | Germany | 2012 | KF577597 |
|  | LRTI-4 | Germany | 2012 | KF938575 |
|  | LRTI-6 | Germany | 2013 | KF577598 |
|  | LRTI-7 | Switzerland | 2013 | KY307857 |
|  | Sibu-97 | Malaysia | 1997 | KY307860 |
|  | VRDL T87-0342 | USA | 1987 | KJ364591 |
|  | LRTI-9 | Switzerland | 2016 | KY307859 |
|  | LRTI-2 | Germany | 2008 | KF802426 |
|  | CDC RU8176 | USA | 1978 | KJ364577 |
|  | NHRC 10030 | USA | 1998 | KJ364586 |
|  | CDC V1375E | USA | 1984 | KJ364579 |
|  | NHRC 20007 | USA | 1998 | KJ364580 |
|  | NHRC 63218 | USA | 2006 | KJ364575 |
|  | NHRC 71139 | USA | 2004 | KJ364583 |
|  | NHRC 71252 | USA | 2005 | KJ364585 |
|  | NHRC 5 | USA | 1996 | KJ364578 |
|  | CDC V2148A | USA | 1988 | KJ364588 |
|  | VRDL T97-1745 | USA | 1997 | KJ364589 |
|  | NHRC 32493 | USA | 2005 | KJ364574 |
|  | VRDL T98-1269 | USA | 1998 | KJ364592 |
|  | GER | Germany |  | KF528688 |
| **SAdV-21** | Bertha | USA | 1954 | AC_000010 |
| **HAdV-B3** | GB | USA | 1953 | AY599834 |
| **HAdV-B7** | Gomen | USA | 1952 | AY594255 |
| **HAdV-B11** | Slobitski | USA | 1956 | NC_011202 |
| **HAdV-B14** | de Wit | Netherlands | 1955 | AY803294 |
| **HAdV-B55** | BJ01 | China | 2011 | JX491639 |
| **HAdV-B34** | Compton | USA | 1972 | AY737797 |
| **HAdV-B35** | Holden | USA | 1973 | AY128640 |
| **HAdV-B16** | ch. 79 | USA | 1955 | AY601636 |
| **HAdV-B50** | Wan | USA | 1988 | AY737798 |
| **HAdV-B66** | AY128640 | Argentina | 1987 | JN860676 |
| **HAdV-B68** | Arg 827/04 | Argentina | 2004 | JN860678 |
| **HAdV-A12** | Huie | USA | 1954 | AC_000005 |
| **HAdV-C1** | Adenoid 71 | USA | 1953 | AF534906 |
| **HAdV-D9** | Hicks | USA | 1954 | AJ854486 |
| **HAdV-E4** | RI-67 | USA | 1952 | AY594253 |
| **HAdV-F40** | Dugan | Netherlands | 1979 | NC_001454 |
| **HAdV-G52** | T03-2244 | USA | 2003 | DQ923122 |

^#^ Isolated in this study.

**Figure S1. Phylogenetic analysis of HAdV-21 strains GZ06109 and GZ09107.** The nucleotide sequences of the hexon (A), penton base (B), and fiber (C) genes of the HAdV strains were analyzed for their phylogenetic relationships using the Neighbor-Joining method with 1,000 bootstrap replicates implemented in the MEGA 11.0.8 software package. For reference, taxon names include the genome type, corresponding GenBank accession number, country of isolation, strain name, and year of isolation. The two HAdV-21 strains isolated in this study are marked with “▲”; “▼”, strain isolated from Bangbu (BB/201903), China in 2019; “●”, reference standard HAdV-21 isolated in Saudi Arabia in 1945.

**Figure S2. Multiple sequence alignment of HAdV-21 major capsid proteins.** Multiple sequence alignment of the full proteins, hexon (A), penton base (B) or fiber (C) from HAdV-21 subtypes 21a, 21b (OHT-006) and 21p (AV-1645), and selected HAdV types. Seven hypervariable regions of hexon are shown. The number shows the position of amino acid in the protein. *****, conserved amino acid; **.**, either size or hydropathy is conserved; and **:**, both size and hydropathy are conserved. Gaps used to optimize alignments are indicated by *dashes.*
