# Supplementary figures and images for: Human Adenovirus Subtype 21a Isolates From Children With Severe Lower Respiratory Illness in China
Source: Front Microbiol. 2022 Jun 16;13:924172. doi: 10.3389/fmicb.2022.924172 (PMC9244545; doi:10.3389/fmicb.2022.924172)

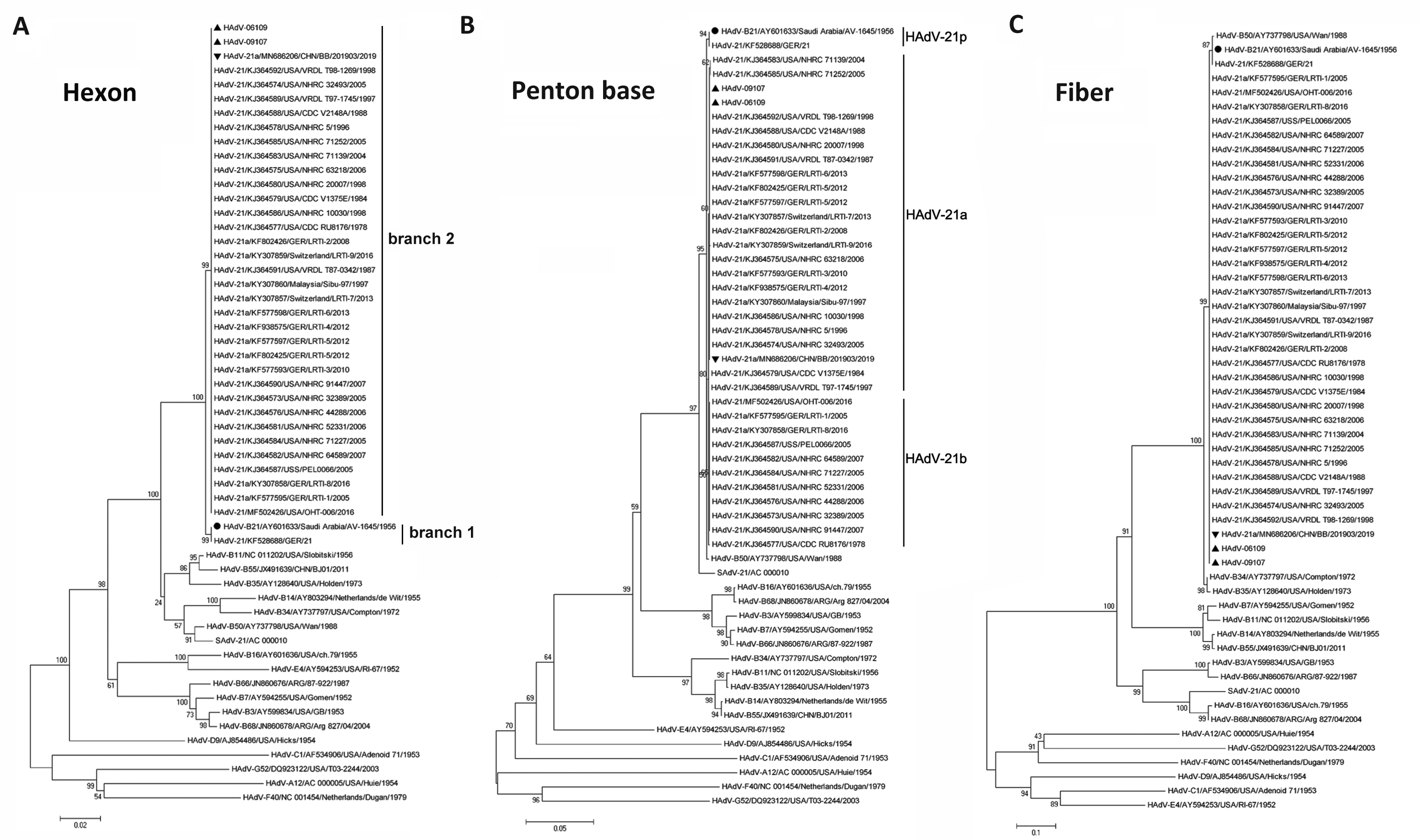

Supplement: Supplementary file 2 [file Image_1.TIF]

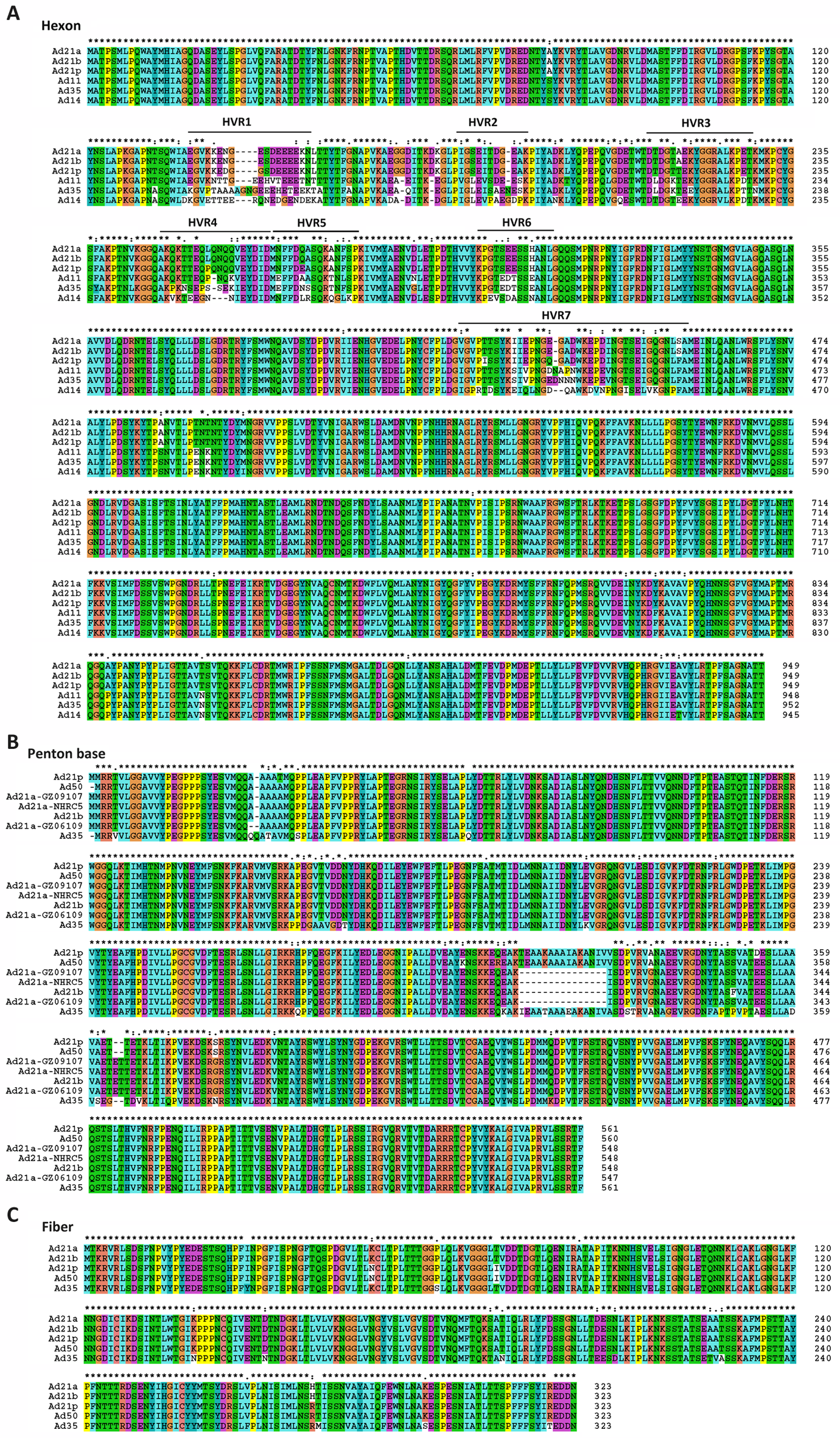

Supplement: Supplementary file 3 [file Image_2.TIF]
